# Neuromuscular responses and inter-limb asymmetry in relation to external load across two sequential pre-season training camp contexts in soccer players

**DOI:** 10.3389/fspor.2026.1860316

**Published:** 2026-07-24

**Authors:** Adam Lipčák, Ana Carolina Paludo, Jan Cacek, Kryštof Cacek, Michal Hrubý, Tomáš Kalina

**Affiliations:** 1 Department of Sport Performance and Exercise Testing, Faculty of Sports Studies, Masaryk University, Brno-Bohunice, Czechia; 2 Department of Psychology, University of Cyprus, Nicosia, Cyprus

**Keywords:** countermovement jump, GPS, hamstring strength, limb dominance, monitoring

## Abstract

**Introduction:**

Different pre-season training camp contexts may be accompanied by distinct neuromuscular responses, including changes in fatigue and inter-limb asymmetry, in soccer players. However, limited research has examined how players’ neuromuscular responses differ across sequential conditioning-focused and game-based camp contexts and how these responses relate to external load.

**Methods:**

Fourteen male national-level soccer players were assessed before and after a 10-day conditioning camp and an 11-day game-based camp separated by 14 days. Neuromuscular performance was evaluated using countermovement jump (CMJ) and Nordic hamstring strength (Nordic Curl), while external load was monitored using GPS-derived metrics, including total distance, high-speed running (HSR), and sprint distance. Percentage changes and correlations between neuromuscular variables and accumulated external load were analyzed using an exploratory descriptive approach.

**Results:**

Following the conditioning-focused camp context, players showed greater neuromuscular changes, including increased CMJ performance (Δ = 3.82%), reduced hamstring strength (Δ = −3.43% and −0.62% for the left and right limbs, respectively), and increased inter-limb asymmetry, whereas only trivial changes were observed following the game-based camp context. Accumulated external load differed between the two camp contexts, with higher total distance and high-speed running during the conditioning-focused camp context and slightly higher sprint distance during the game-based camp context. Relationships between external load and neuromuscular changes differed between camps. During the conditioning camp, higher external load was associated with a smaller increase in asymmetry, whereas during the game-based camp, external load was related to reductions in CMJ performance and increases in hamstring strength.

**Conclusion:**

Players showed different neuromuscular responses across the two sequential camp contexts, and load–response relationships appeared to be context dependent and individual. Given the small sample and exploratory design, these findings should be interpreted cautiously. Integrating external load monitoring with neuromuscular assessments may improve training and recovery strategies in team sports.

## Introduction

Monitoring training load and its impact on neuromuscular performance has become a key component of performance optimization and injury prevention in team sports. External load, commonly assessed using GPS-derived metrics such as total distance, high-speed running, and sprint distance, provides valuable information about the physical demands imposed on players (1). At the same time, neuromuscular assessments, including countermovement jump (CMJ) performance and hamstring strength, are widely used to evaluate fatigue, readiness, and injury risk (2, 3).

Previous research has shown that accumulated training and match load can influence neuromuscular performance, often leading to transient reductions in force production and power output due to fatigue (4, 5). However, the relationship between external load and neuromuscular responses is not always consistent, with some studies reporting performance maintenance or even improvements following specific training stimuli. These discrepancies suggest that neuromuscular responses to load may depend on factors such as training context, intensity, and individual player characteristics (6).

In addition to performance measures, inter-limb asymmetry has gained increasing attention as a potential indicator of neuromuscular imbalance and injury risk. Previous studies have suggested that asymmetry may be influenced by fatigue and training load, although the direction and magnitude of these changes remain unclear (2, 7). Furthermore, most existing research has focused on isolated training periods or match play, with limited attention given to how different training contexts may influence asymmetry and neuromuscular responses.

Importantly, different types of training camps, such as conditioning-focused and game-based camps, may impose different load profiles and physiological demands on players. In the present study, a conditioning-focused camp refers to a training context emphasizing physical development, strength training, conditioning drills, and small-sided games, whereas a game-based camp refers to a context emphasizing tactical preparation and match exposure. Conditioning camps are typically characterized by higher training volume and structured physical development, whereas game-based camps emphasize tactical preparation and match exposure (8). Despite these differences, little is known about how these contexts influence neuromuscular responses and their relationship with external load.

Therefore, the aim of this exploratory study was to investigate changes in neuromuscular performance (CMJ and hamstring strength), inter-limb asymmetry, and their relationship with external load, across two sequential pre-season training camp contexts in soccer players. Rather than testing a confirmatory hypothesis, the study explored whether players would show greater neuromuscular changes following the conditioning-focused camp context, including fatigue-related responses and inter-limb asymmetry, compared with the game-based camp, and whether external load would be related to changes in neuromuscular variables.

## Methods

### Study design

**Figure 1:**
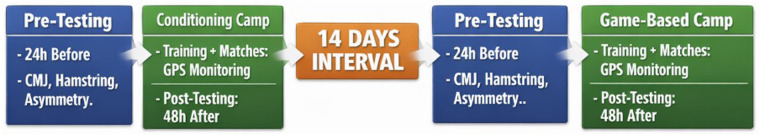
Overview of the study design and training camp structure.

This study employed an exploratory observational repeated-measures design to investigate neuromuscular responses and inter-limb asymmetry in soccer players across two different real-world pre-season training camps (conditioning-focused and game-based). The conditioning camp lasted 10 days, the game-based camp lasted 11 days, and the two camps were conducted at two separate time points with a 14-day interval between them. The same group of players was followed across both camps; therefore, the camps should be interpreted as two applied training contexts rather than as independent randomized interventions. Neuromuscular performance, including CMJ, hamstring strength, and inter-limb asymmetry, was assessed before and after each training camp. External load was continuously monitored during both camps using GPS-derived metrics, including total distance, high-speed running, and sprint distance (Figure 1). The percentage change (Δ %) between pre- and post-camp measurements was calculated for all neuromuscular variables. These changes were subsequently analyzed in relation to the accumulated external load during each camp. Additionally, descriptive comparisons were made across the two sequential camp contexts to examine differences in players' neuromuscular responses and load-response relationships.

### Participants

Fourteen male national-level soccer players (mean ± SD, age: 28.29 ± 4.60 years; height: 182 ± 6.31 cm; body mass: 81.01 ± 5.06 kg) from a first division soccer team in the Czech Republic participated in this study. All participants were regularly competing and engaged in structured training programs. Although the full squad included more players, only players who completed all training sessions, match exposures, and all pre- and post-camp testing sessions across both camps were included in the final analysis. Players with incomplete training, match, or testing data were excluded to ensure that all analysed players had comparable exposure and complete repeated-measures data. Inclusion criteria required players to be free from injury at the time of testing and to have no history of lower-limb injuries in the preceding 6 months. Exclusion criteria included any current injury or musculoskeletal condition that could affect performance during testing. All participants were informed about the procedures and provided written informed consent prior to participation. The study protocol was approved by the Masaryk University Research Ethics Committee (approval number: EKV-2024-131), and the study was conducted in accordance with institutional ethical requirements for research involving human participants.

### Testing procedures

All testing procedures were conducted in the morning, at the beginning of a new microcycle, and before the first scheduled training session of the respective camp. Pre-camp assessments were performed 24 h before the start of each training camp, while post-camp assessments were conducted 48 h after the end of each camp. This timing was selected to standardize the testing schedule and to reduce the influence of acute fatigue immediately after the final camp exposure. However, the external load accumulated during the days preceding the pre-camp assessments, particularly before the game-based camp, was not quantified separately. Prior to testing, all participants completed a standardized warm-up consisting of mobility exercises and dynamic stretching. Neuromuscular testing was performed in a fixed order, starting with the countermovement jump (CMJ), followed by the Nordic hamstring exercise (Nordic Curl) to assess hamstring strength.

#### Training camp characteristics

Two different training camps were analysed: a conditioning-focused camp and a game-based camp. Both camps were conducted during the pre-season period. The conditioning camp was performed first and represented a conditioning-focused microcycle held in Turkey. The game-based camp was conducted 14 days later and took place in Portugal. During the 14-day interval between the camps, players continued with their regular team training, which consisted of standard pre-season sessions without any specific experimental intervention. The load accumulated during this inter-camp period was not included in the camp-specific external load analysis, and possible residual or carry-over effects from this period were therefore considered when interpreting the findings.

The conditioning camp lasted 10 days and included 11 training sessions and 2 matches. Training content primarily consisted of strength training, conditioning drills, and small-sided games, with an emphasis on physical development. The game-based camp lasted 11 days and included 8 training sessions and 3 matches. This camp was characterized by a higher frequency of matches, a greater focus on tactical preparation, and a lower overall training volume. External load was monitored during all training sessions and matches in both camps using GPS devices. All analysed players completed all training sessions, matches, and pre- and post-camp assessments included in the study period; therefore, player availability was handled as an inclusion criterion rather than as a separate outcome.

### Neuromuscular testing

#### Countermovement jump

Countermovement jump performance was assessed using a dual force plate system (ForceDecks, FDLite V.2, VALD, Brisbane, Australia). Players performed three maximal CMJ trials, with hands placed on the hips to eliminate arm swing. Each jump was executed following a rapid downward movement immediately followed by a maximal vertical propulsion. A 60 s rest interval was provided between trials to minimize fatigue. Jump height (cm) was used for analysis, and the best trial was retained for further statistical analysis. The best trial was selected because the aim was to characterize maximal neuromuscular capacity under standardized testing conditions and to reduce the influence of submaximal attempts or pacing strategies.

#### Hamstring strength assessment

Hamstring strength was assessed using the Nordic hamstring exercise performed on a NordBord device (Vald Performance NordBord, Version 1.0, Australia) (9). Players completed three maximal trials while resisting forward movement as long as possible, maintaining a controlled descent. Peak force (N) values were recorded for each limb, and the highest value obtained for each limb was used for further analysis. Consistent with the CMJ procedure, the highest value was retained to represent maximal eccentric hamstring strength capacity for each limb.

#### Inter-limb asymmetry calculation

Inter-limb asymmetry was calculated based on hamstring peak force values using the method described by (10), according to the following formula:

$$\mathrm{Asymmetry}(\text{\%})=\frac{100}{\mathrm{maxvalue}\times \mathrm{minvalue}}\times -1+100$$

Absolute asymmetry values were used for subsequent analyses.

### External load monitoring

External load was monitored using an 18 Hz GPS tracking system STATSports Apex 2.0 (STATSports Apex, Northern Ireland) (11). All players wore the same GPS unit throughout the study period to minimize inter-unit variability. External load variables included total distance (TD), high-speed running distance (HSR), and sprint distance (SD), which were recorded during all training sessions and matches in both camps. High-speed running was defined as distance covered at speeds between 19.8 and 25.2 km·h^−1^, while sprint distance was defined as distance covered at speeds grear than 25.2 km·h^−1^. All external load metrics were accumulated across each training camp for further analysis. These GPS-derived variables were used as indicators of external load volume and intensity; training and match exposure time in minutes was not examined separately.

### Data processing

The percentage change (Δ %) between pre- and post-camp measurements was calculated for CMJ and hamstring strength (left and right limbs) using the following formula:

$$\Delta \text{\%}=\left(\frac{\mathrm{post}-\mathrm{pre}}{\mathrm{pre}}\right)\times 100$$

Inter-limb asymmetry changes were analysed as the absolute difference between post- and pre-camp values (post − pre), expressed in percentage points. External load variables (total distance, high-speed running, and sprint distance) were accumulated across each training camp for each player. Individual changes in neuromuscular variables were then used to examine their relationships with accumulated external load using correlation analysis.

### Statistical analysis

Descriptive statistics are presented as mean ± standard deviation (SD). The normality of the data distribution was assessed using the Shapiro–Wilk test. Changes in neuromuscular variables were quantified using percentage change (Δ %) for CMJ and hamstring strength, while inter-limb asymmetry was expressed as the absolute difference between post- and pre-camp values (percentage points). Given the small sample size and the applied observational design, the statistical approach was primarily descriptive and exploratory. Inferential tests for pre- to post-camp comparisons were not performed; therefore, these changes should be interpreted as descriptive within-sample responses rather than confirmatory evidence of camp effects. Effect sizes (ES; Cohen's *d*) were calculated to assess the magnitude of changes and were interpreted as trivial (<0.2), small (0.2–0.5), moderate (0.5–0.8), large (>0.8) (12). Cohen's *d* thresholds were retained because the reported effect sizes were calculated using Cohen's *d* and were intended to support descriptive interpretation of the magnitude of change. Pearson's correlation coefficients were used to examine the relationships between changes in neuromuscular variables and accumulated external load metrics. Correlation analyses were also considered exploratory. No correction for multiple comparisons was applied because the aim was to describe potential load-response patterns; consequently, correlation coefficients and *p*-values should be interpreted cautiously and not as definitive evidence of statistically robust associations. All statistical analyses were performed using Python (Python Software Foundation, Wilmington, DE, USA). The level of statistical significance was set at *p* < 0.05.

## Results

Neuromuscular changes across both training camps are presented in Table 1. Descriptively, following the conditioning-focused camp context, CMJ performance increased (Δ = 3.82%, ES = 0.69), while hamstring strength decreased in both the left (Δ = −3.43%, ES = −0.42) and right limb (Δ = −0.62%, ES = −0.12). Inter-limb asymmetry increased (Δ = 3.39%, ES = 0.44). In contrast, smaller changes were observed following the game-based camp context, with trivial increases in CMJ performance (Δ = 1.24%, ES = 0.15) and minimal changes in hamstring strength and asymmetry.

**Table 1:** Neuromuscular and asymmetry changes across Two sequential training camps contexts.

Camp	Variable	Mean Pre ± SD	Mean Post ± SD	ES	Changes [%]	Absolute Change
Conditioning	CMJ [cm]	38.09 ± 4.6	39.44 ± 4.3	0.69	3.82	+1.35
	Nordic Left [N]	414.29 ± 54.93	399.86 ± 62.78	−0.42	−3.43	−14.43
	Nordic Right [N]	430.79 ± 45.73	425.86 ± 44.41	−0.12	−0.62	−4.93
	Asym [%]	7.72 ± 5.93	11.11 ± 8.59	0.44	3.39	+3.39
Game-Based	CMJ [cm]	37.79 ± 3.73	38.19 ± 3.85	0.15	1.24	+0.40
	Nordic Left [N]	409.07 ± 66.78	401 ± 62.56	−0.2	−1.31	−8.07
	Nordic Right [N]	417.43 ± 52.32	419 ± 36.51	0.05	1.05	+1.57
	Asym [%]	7.24 ± 7.75	8.83 ± 7.14	0.21	1.59	+1.59

SD, standard deviation; ES, effect size; CMJ, countermovement jump; Nordic, nordic curl; Asym, asymmetry; cm, centimetre; N, newton.

External load variables for both training camps are presented in Table 2. The conditioning-focused camp context was characterized by higher accumulated total distance and high-speed running compared with the game-based camp context. In contrast, sprint distance was slightly higher during the game-based camp context.

**Table 2:** External load during conditioning and game-based training camps.

Camp		Total distance [m]	HSR [m]	Sprint distance [m]
Conditioning	Mean ± SD	71,172 ± 5,067	2,601 ± 909	426 ± 304
Game-Based	Mean ± SD	55,482 ± 6,930	2,445 ± 525	466 ± 168

SD, standard deviation; HSR, high speed running; m, meter.

**Figure 2:**
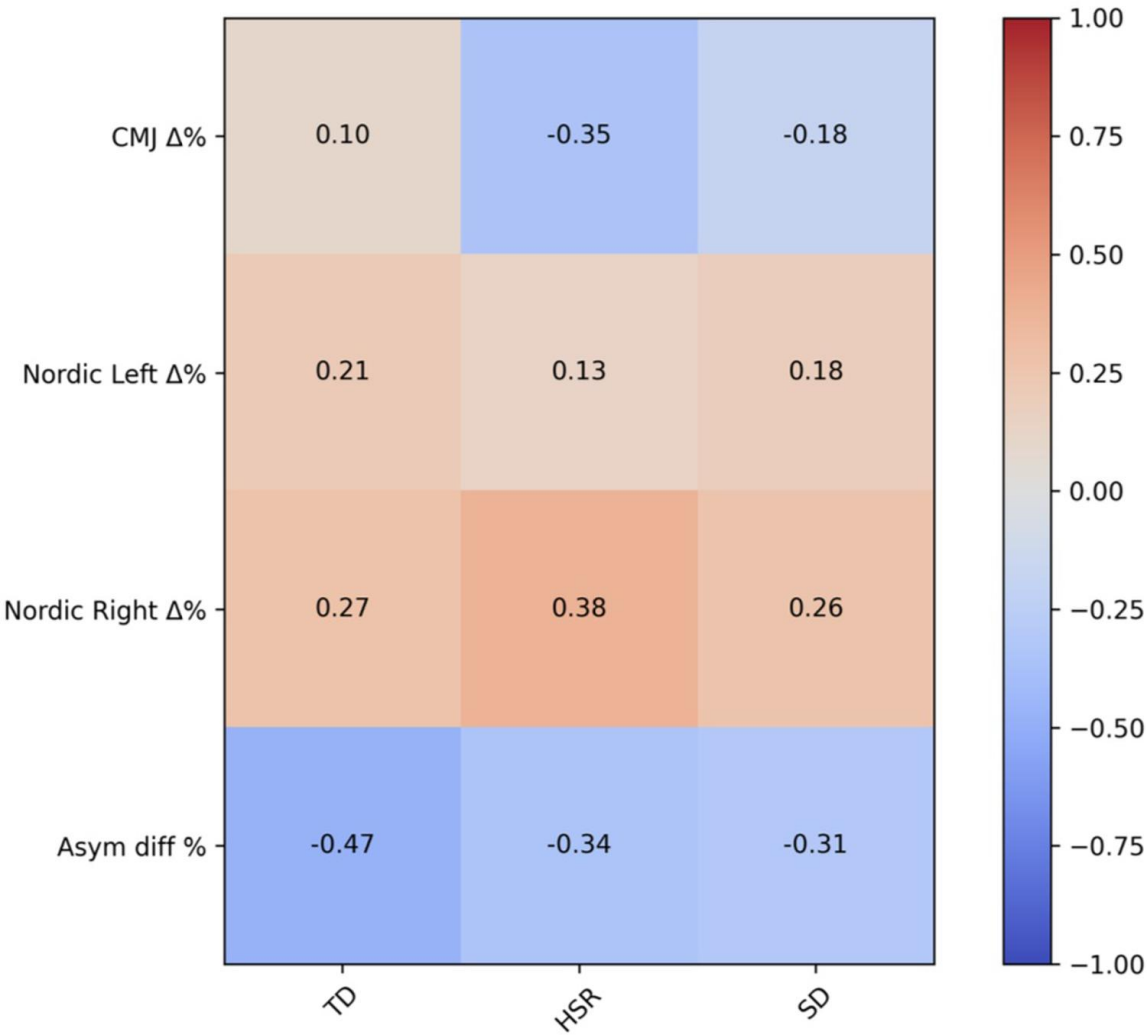
Correlation matrix between neuromuscular changes and external load variables during the conditioning camp. Δ%, delta percentage; CMJ, countermovement jump; Nordic, nordic curl; Asym diff, asymmetry differences; TD, total distance; HSR, high speed running; SD, sprint distance.

Exploratory correlations between neuromuscular changes and external load variables during the conditioning-focused camp context are presented in Figure 2. A moderate negative relationship was observed between total distance and changes in inter-limb asymmetry (*r* = −0.47, *p* = 0.09). Similar negative associations were found between asymmetry changes and both high-speed running (*r* = −0.34, *p* = 0.23) and sprint distance (*r* = −0.31, *p* = 0.28). CMJ changes showed trivial to small correlations with external load variables. Positive relationships were observed between hamstring strength changes and external load, with the strongest association found between right limb hamstring strength and high-speed running (*r* = 0.38, *p* = 0.18).

**Figure 3:**
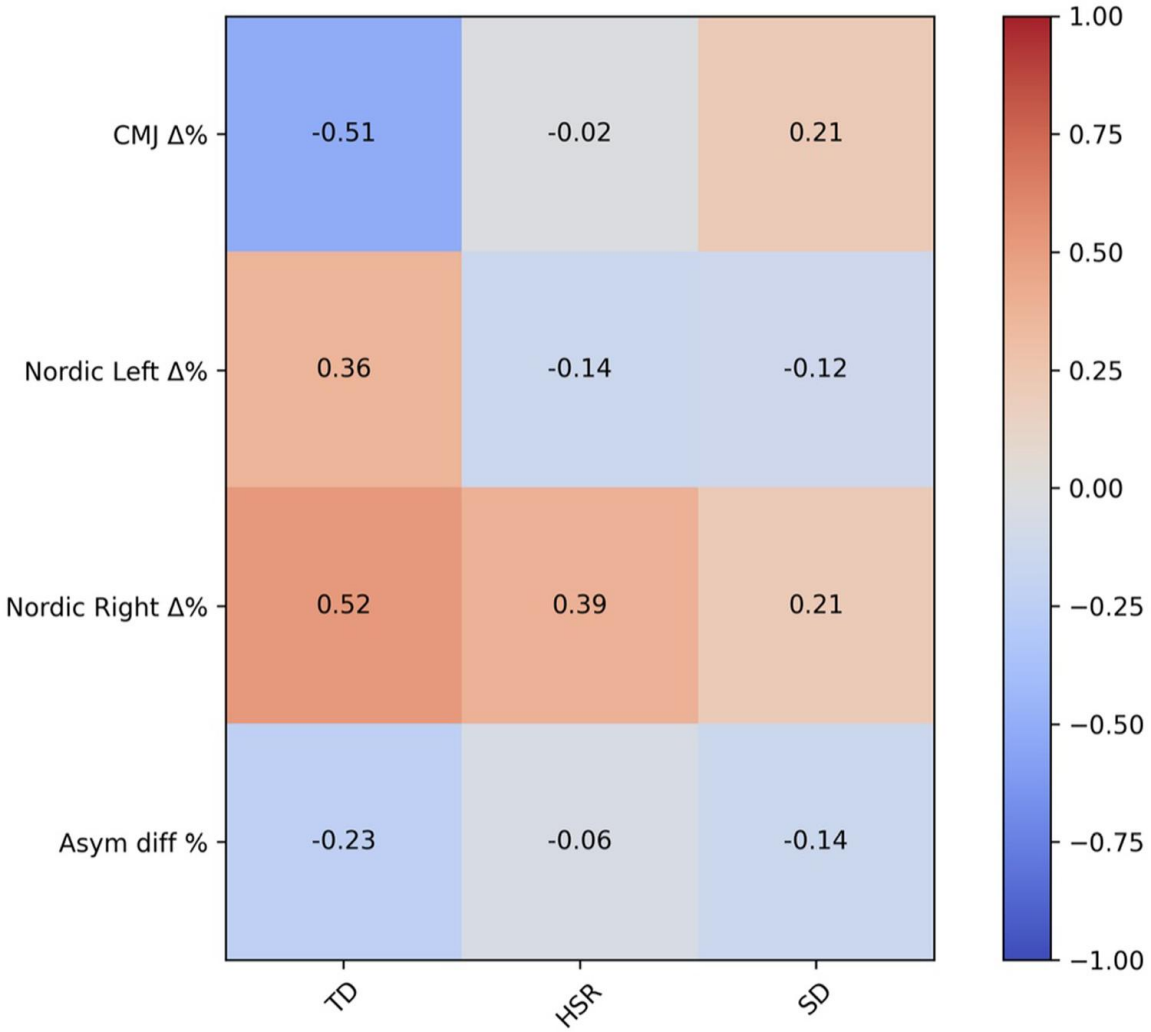
Correlation matrix between neuromuscular changes and external load variables during the game-based camp. Δ%, delta percentage; CMJ, countermovement jump; Nordic, nordic curl; Asym diff, asymmetry differences; TD, total distance; HSR, high speed running; SD, sprint distance.

Exploratory correlations between neuromuscular changes and external load variables during the game-based camp context are presented in Figure 3. A moderate negative relationship was observed between total distance and changes in CMJ performance (*r* = −0.51, *p* = 0.06). Positive correlations were found between hamstring strength changes and external load, with the strongest association observed between right limb hamstring strength and total distance (*r* = 0.52, *p* = 0.06), followed by high-speed running (*r* = 0.39, *p* = 0.17). Changes in inter-limb asymmetry showed trivial to small negative relationships with external load variables.

## Discussion

The aim of this exploratory study was to investigate changes in neuromuscular performance and inter-limb asymmetry, and their relationship with external load, across two sequential pre-season training camp contexts in soccer players. The main descriptive findings were that players showed greater changes following the conditioning-focused camp context in neuromuscular variables, including increases in CMJ performance and inter-limb asymmetry, alongside reductions in hamstring strength. In contrast, only trivial changes in neuromuscular performance were observed following the game-based camp context. Furthermore, the exploratory relationships between external load and neuromuscular changes differed between camps. During the conditioning camp, higher external load was associated with reductions in inter-limb asymmetry, while during the game-based camp, larger correlation coefficients were observed between external load and changes in hamstring strength and CMJ performance. Because the game-based camp occurred 14 days after the conditioning camp while players continued regular team training, the observed responses cannot be attributed solely to camp type. Instead, they should be interpreted as responses to two sequential pre-season contexts, with possible influence of inter-camp training stimuli and recovery status.

### Neuromuscular changes

The present study showed that neuromuscular responses differed across the two sequential camp contexts, with greater changes observed following the conditioning camp. Specifically, CMJ performance increased, while hamstring strength decreased and inter-limb asymmetry increased.

The increase in CMJ performance observed after the conditioning-focused camp context may reflect a potentiation effect or short-term neuromuscular adaptation associated with the accumulated training stimulus. Such responses have been previously reported following periods of intensified training or mixed training stimuli combining strength and high-intensity efforts (13). In contrast, the observed reduction in hamstring strength may indicate the presence of residual neuromuscular fatigue, particularly in response to repeated high-intensity actions such as sprinting and eccentric loading during training and matches (14, 15). The Nordic hamstring exercise has been shown to be sensitive to fatigue-related changes in eccentric strength capacity (9).

Additionally, the increase in inter-limb asymmetry observed after the conditioning camp may reflect an imbalance in neuromuscular function induced by accumulated fatigue or uneven load distribution between limbs (2). Previous research has suggested that fatigue can exacerbate asymmetries, potentially increasing injury risk or reducing movement efficiency (16). In contrast, only trivial changes were observed after the game-based camp context in neuromuscular variables, which may be explained by the lower overall training volume and greater emphasis on tactical and match-related activities. These findings suggest that different training contexts can lead to different neuromuscular responses, even when overall exposure to match play is similar (6).

### External load and neuromuscular responses

The present study showed that exploratory relationships between external load and neuromuscular changes differed across the two sequential camp contexts, highlighting the context-dependent nature of load–response interactions.

During the conditioning-focused camp context, higher external load was associated with a smaller increase in inter-limb asymmetry, as indicated by negative correlations between total distance, high-speed running, sprint distance, and asymmetry changes. However, despite these relationships, inter-limb asymmetry increased at the group level following the conditioning camp. This suggests that while accumulated training load may contribute to an overall increase in asymmetry, players exposed to higher loads may experience less pronounced negative changes. These findings highlight the complexity of load–response relationships and suggest that individual responses to training load may differ considerably (17, 18). In contrast, during the game-based camp context, external load showed larger correlations with changes in CMJ performance and hamstring strength. Specifically, higher total distance was related to reductions in CMJ performance, which may reflect neuromuscular fatigue induced by accumulated match and training load (4). At the same time, positive relationships were observed between external load and hamstring strength, particularly in the right limb, suggesting a load-induced neuromuscular stimulus or a protective adaptation to repeated high-intensity actions such as sprinting (19, 20).

Overall, these findings suggest that the association between external load and neuromuscular performance was not uniform across players or camp contexts but instead elicits variable responses depending on the training context and the specific neuromuscular variable assessed. This supports the notion that load–response relationships in team sports are highly individual and context-specific, emphasizing the importance of integrating both external load and neuromuscular monitoring when evaluating player readiness and fatigue (3, 6).

### Limitations

Several limitations of this study should be acknowledged. First, the relatively small sample size may limit the generalizability of the findings. The final sample of 14 players resulted from strict inclusion criteria requiring complete participation in all training sessions, matches, and testing sessions across both camps. Although this improved consistency of exposure within the analysed sample, it reduced statistical power and limits the extent to which the findings can be generalized to the full squad or other teams. However, the sample consisted of national-level soccer players, which enhances the ecological validity of the results and reflects the practical difficulty of conducting repeated neuromuscular assessments in professional football environments. Second, the exploratory observational repeated-measures design does not allow for causal inferences between external load and neuromuscular changes, and the absence of two independent groups or repeated observations across different seasons limits the ability to separate camp effects from broader pre-season effects. Third, the two camps were separated by a 14-day period during which players maintained regular team training stimuli. Because the external load, internal load, and recovery status during this inter-camp period were not quantified, residual fatigue, adaptation, or carry-over effects may have influenced the pre-camp values before the game-based camp. Fourth, only selected external load variables were considered. Training and match exposure time in minutes and time-loss injury incidence during each camp were not analysed as separate outcomes, while player availability was handled through the inclusion criteria. Fifth, the conditions preceding the pre-camp assessments were standardized by scheduling testing at the beginning of a microcycle and before camp exposure, but the load accumulated in the preceding days and the number of recovery days before each pre-test were not fully controlled. Sixth, no inferential pre- to post-camp tests or corrections for multiple correlations were applied. Therefore, the reported effect sizes, percentage changes, and correlation coefficients should be interpreted as exploratory indicators of possible response patterns rather than confirmatory evidence. Finally, individual variability in responses to training load may have influenced the observed relationships, highlighting the need for individualized monitoring approaches in future research.

### Practical applications

The findings of this study provide several practical implications for coaches and practitioners working in team sports. First, the results highlight that players may show different neuromuscular responses across different training camp contexts, suggesting that conditioning-focused periods may be accompanied by greater neuromuscular stress than game-based contexts, although this should not be interpreted as a causal effect of camp type. Second, the observed dissociation between CMJ performance and hamstring strength emphasizes the importance of using multiple neuromuscular assessments, as relying on a single measure may not fully capture the fatigue or readiness status of athletes. Furthermore, the relationship between external load and neuromuscular changes reinforces the need for integrated monitoring approaches combining GPS-derived metrics with neuromuscular testing to better understand individual responses to training. In applied settings, practitioners should also document training and match exposure in minutes, completion of planned training and match exposure, injury occurrence, and recovery days before testing, as these contextual factors may influence neuromuscular responses and the interpretation of load-response relationships. Finally, the variability in individual responses suggests that practitioners should adopt individualized monitoring strategies rather than relying solely on group averages when managing training load and recovery.

## Conclusion

In this exploratory observational study, players showed different neuromuscular responses across two sequential pre-season training camp contexts. Greater changes were observed following the conditioning-focused camp context, including increased CMJ performance, reduced hamstring strength, and increased inter-limb asymmetry, whereas only minimal neuromuscular alterations were observed following the game-based camp context. Exploratory relationships between external load and neuromuscular changes also differed across the two sequential camp contexts, highlighting the context-dependent and individual nature of load–response interactions. Given the small sample size and descriptive analytical approach, these findings should be interpreted cautiously and used to inform future studies with larger samples, repeated seasons, and more complete load-response monitoring. These findings emphasize the importance of integrating external load monitoring with neuromuscular assessments to better understand athlete readiness and fatigue, and to support more individualized training and recovery strategies.

## Data Availability

The original contributions presented in the study are included in the article/Supplementary Material, further inquiries can be directed to the corresponding author.
